# Trust in leadership and perceptions of justice in fostering employee commitment

**DOI:** 10.3389/fpsyg.2024.1359581

**Published:** 2024-01-31

**Authors:** Alejandro González-Cánovas, Alejandra Trillo, Francisco D. Bretones, Juan M. Fernández-Millán

**Affiliations:** ^1^Faculty of Social Sciences and Law, University of Granada, Melilla, Spain; ^2^Faculty of Labor Relations and Human Resources, University of Granada, Granada, Spain

**Keywords:** authentic leadership, affective commitment, distributive justice, interactional justice, trust

## Abstract

In a period of uncertainty, trust in leadership and perceptions of fairness have emerged as pivotal factors for fostering employee identification and affective commitment. Drawing from authentic leadership theory, this leadership style is identified as a crucial antecedent of affective commitment, examining the mediating role of distributive justice and the moderating role of interactional justice. A quantitative approach was employed, utilizing data from 302 questionnaires completed by Spanish retail workers. For data analysis, SPSS v.25 was used to generate descriptive statistics, while partial least squares structural equation modeling was applied to test the proposed hypotheses. Our findings revealed that authentic leadership is positively associated with the development of affective commitment, with distributive justice acting as a mediating factor between the two. Furthermore, interactional justice negatively moderates the relationship between distributive justice and affective commitment. Contrary to initial expectations, the second moderation, between authentic leadership and affective commitment, was not found to be significant. The research concludes by discussing the practical implications of the results.

## 1 Introduction

Current organizational environments, marked by complexity, high volatility, and uncertainty, are prompting trust in leaders and perceptions of justice to become highly valued by workers. Indeed, their effectiveness is partly based on an implicit agreement of reciprocity between the organization and its employees. Hence, supervisors play a crucial role in creating trusting environments that can lead to an increase in employees’ identification and commitment towards the organizations they work for.

The idea of the existence of a reciprocal relationship between supervisor and employee has been raised by some authors (Leroy et al., 2012; Alfes et al., 2013) who, drawing on Blau’s (1964) Social Exchange Theory, suggest that relationships between workers and their leaders are characterized by dynamic interactions seeking reciprocal and trustworthy behaviors, ultimately leading to mutual commitment. Consequently, when workers perceive these values from their leaders, they feel indebted, a sentiment that enhances their commitment to the organization (Xiong et al., 2016).

However, in practice, the presence of attitudes that are contrary to ethics and authenticity among some leaders hinders the achievement of this reciprocity. As a result, It is necessary to include leadership styles that include moral and ethical aspects, such as Authentic Leadership (AL). This theory is introduced as a more constructive approach to organizational leadership that can restore employees’ trust at various leadership levels and promote a work culture of positive attitudes and behaviors (Gill et al., 2018). Although the concept of authentic leadership is diversified into multiple definitions, all of them share fundamental values (behavioral integrity, respect for self-assessment, and authenticity), which are unique and differentiate them from other leadership styles (Lemoine et al., 2019).

Walumbwa et al. (2008) identified four basic dimensions of authentic leadership: self-awareness (referring to the ability to recognize strengths and limitations); relational transparency (ability to encourage the expression of ideas and feelings); balanced processing of information and measured judgment before making decisions; and internalized morality as a guide to values and personal principles in the face of external influences. Thus, AL is defined as a leadership behavior characterized by clear communication, self-awareness, and balanced judgment, focused on the common good and the development of employees (Zeb et al., 2020).

A distinctive feature of authentic leadership (AL) is its ability to influence and generate trust among its followers. In this regard, Legood et al. (2020), through a meta-analysis, determined that AL generated a higher degree of cognitive trust (34.92%) compared to other leadership styles. Consequently, various authors have asserted that authentic leaders can induce favorable behaviors in their employees, which has a positive impact on organizational performance and, specifically, on organizational commitment (AC) (Semedo et al., 2016; Hashim et al., 2017; Ribeiro et al., 2018).

Moreover, in an era where corporate corruption is unfortunately becoming more common news, stakeholders in organizations (including the companies’ own workers) demand leaders who act with high integrity and fairness (Walumbwa et al., 2008). Previous research has demonstrated that authentic leaders possess the quality of developing and maintaining strong relationships based on trust and make logical decisions based on values, fostering a perception of fairness among organization members (Alinezhad et al., 2015; Divya and Suganthi, 2018; Kurian and Nafukho, 2022). However, numerous literature reviews reveal the need for more empirical research to deeply understand the authentic leader-follower relationship (Gardner et al., 2011; Avolio and Walumbwa, 2014; Alilyyani et al., 2018), as well as the influence of other variables in this relationship.

With this study, we aim to make some important contributions. First, by analyzing how authentic leadership can influence affective commitment, we provide deeper understanding into the way a leader’s qualities can cause emotional resonance and dedication among employees. In doing so, we fill a gap in the current research, which frequently focuses more on organizational outcomes or performance rather than on the emotional responses of its members. Additionally, while previous studies have considered the intermediating and moderating role of organizational justice in the relationship between AL and AC, our research has selected two of its dimensions to clarify under what conditions AL is more likely to foster affective commitment, as well as the situations that may limit or enhance its impact.

In the following sections, we present the literature review and the hypotheses underpinning our research. Subsequently, we will outline the methodology followed in the study and then describe the findings obtained. Finally, we present the discussion, the contributions of the study, as well as its limitations and future research areas.

## 2 Literature review

### 2.1 Authentic leadership and affective commitment

Leaders play a crucial role in fostering the affective commitment as previous research with various leadership approaches has demonstrated (Jeon and Choi, 2020; Bai et al., 2023). Specifically, the effect of authentic leadership has been examined as these leaders are more adept at cultivating affective bonds with subordinates, thereby deepening the affective commitment these employees develop towards their workplace (Rego et al., 2016; Hadian Nasab and Afshari, 2019). Consequently, authors like Schoorman et al. (2007) argue that when leaders conduct themselves with transparency, they reduce ambiguity and risk associated with interactions with them, crafting an environment where they are perceived as both integral and capable by their employees.

On the other hand, existing research reveals that, of the three factors of organizational commitment that make up the multidimensional model proposed by Meyer and Allen (1997), the affective dimension has the greatest bearing on individual behavior within the workplace environment. This is because it represents an approach towards the organization itself, whereas normative and continuous commitment are approaches towards specific kinds of behavior (Solinger et al., 2008; Ribeiro et al., 2020). This evolution has prompted many scholars to study this type of commitment more extensively in recent years, with its description gradually developing. Moreover, the concept of Organizational Commitment (AC) is one of the most studied topics in the field of human resources, both for its impact on employee well-being and for its influence on performance, retention, and talent management (Kanste, 2011; Mercurio, 2015).

Therefore, it is very likely that the trust that the authentic leader generates in his followers will be transformed into attachment and identification with the organization, ultimately fostering greater affective commitment. Considering these antecedents, we propose the following hypothesis:

*H1*: Employees' perceptions of Authentic Leadership (AL) will positively influence their Affective Commitment (AC) towards the organization.

### 2.2 The mediating role of distributive justice

Leaders’ behavior has an impact on other perceptual variables. A review by Karam et al. (2019) suggests that perceptions of justice between leaders and peers (i.e., supervisor-focused justice) analyzed how perceptions of fairness between leaders and coworkers, regarding supervision, influence employee results more than other organizational views (Shao et al., 2022). Similarly, in different work areas, the thought of receiving what’s deserved compared to others’ pay can affect employee commitment. This type of distributive justice has been defined as a set of perceptions that employees hold about what is fair or equitable in a company concerning decisions related to the allocation of resources or rewards (Niehoff and Moorman, 1993).

The role of the leader in this process is crucial, as they can modulate employees’ perceptions of justice (Kurian and Nafukho, 2022). In the case of authentic leadership style, when supervisors present themselves as trustworthy and equitable, they instill in their teams a vision of the organization as more egalitarian and reliable (Jabbouri, 2021).

Therefore, drawing upon the existing literature, we propose the following research hypothesis:

*H2*: Perception of Authentic Leadership (AL) has a positive impact on the level of Distributive Justice (DJ).

Additionally, organizational justice has other organizational impacts, particularly on organizational commitment (Kassim and Ibrahim, 2016; Swalhi et al., 2017). This relationship may be attributed to the fact that when a company rewards its employees fairly, they may perceive this not only as a remunerative act but also as a mutual commitment, wherein the employee, in response to the company’s care and attention, develops affection and commitment towards it (Powell and Meyer, 2004). Other authors such as Cropanzano et al. (2007) suggest different reasons why employees with a higher perception of distributive justice tend to develop a stronger affective commitment to their organization. This is because it fosters a greater sense of control, thereby reducing uncertainty about future income, and may reflect a higher status within the organization.

Considering these antecedents, we propose the following hypothesis:

*H3*: Positive perceptions of Distributive Justice (DJ) will have a positive impact on employees' Affective Commitment (AC).

However, Distributive Justice (DJ) not only impacts Affective Commitment (AC) but may also mediate the influence of leadership on the latter.

Recall that empirical evidence has demonstrated how authentic leadership leads to a positive perception of justice among subordinates, which in turn generates proactive responses characterized by positive attitudes towards the organization. In this regard, various authors (Kiersch and Byrne, 2015; Primawidi and Mangundjaya, 2020) have indicated that organizational justice could play a mediating role in authentic leadership, given the significance of integrity and moral virtue inherent in authentic leadership (Folger et al., 2013). Additionally, employees’ perceptions of justice significantly influence their attitudes towards the organization, particularly impacting Affective Commitment (AC). This is because fair treatment by the organization results in increased self-esteem and the feeling of value in a reciprocal relationship, which leads to employee commitment, as has been demonstrated in various studies (Lee and Wei, 2017; O’Connor and Crowley-Henry, 2019).

Thus, the following hypothesis is:

*H4*: The perception of Distributive Justice (DJ) mediates the relationship between Authentic Leadership (AL) and Affective Commitment (AC).

### 2.3 The moderating role of interactional justice

But distributive justice or injustice is perceived when employees feel they are paid less, not because they are not less valuable to the organization or because they are less important, but because of poor organizational performance (Jiang and Law, 2013).

Interactional Justice (IJ) refers to the view of interpersonal treatment that employees get from the company and its leaders (Murtaza et al., 2011). The significance of IJ lies in the fact that, unlike distributive justice, which is based on resource exchange, it does not depend on such allocation, but rather has a more every day and affective implication (Li et al., 2017). Thus, when employees perceive that the executives of the organization exhibit fair behavior towards them, they will respond diligently to their work and exhibit behavior indicative of job commitment (Kerse and Naktiyok, 2020).

This can be corroborated by examining the symbolic component of social exchange, where the fulfilment of promises made by authority to subordinates reinforces the valuation of that employee in the collective. Therefore, the notion that maintaining high levels of interactional justice over time can result in social exchanges between employees and their supervisors, thus fostering a psychological contract between employees and the organization, could have a positive effect on the relationship between distributive justice and positive organizational attitudes. Whereby, even in situations of distributive injustice, the impact of this injustice on workers’ attitudes could be partially mitigated by the presence of interactional justice (Ramkissoon, 2016), which would have a moderating rather than mediating role in this relationship.

Therefore, drawing on the existing literature and conceptualizing Interactional Justice as a potential moderator, we formulate the following hypothesis:

*H5a*: Interactional Justice (IJ) moderates the effect of Distributive Justice (DJ) on Affective Commitment (AC).

An employee’s perspective of how they interact and connect with their direct managers will also be influenced by how approachable and understanding their line managers seem. When leaders act in a trustworthy and genuine way, greater belief in equitable treatment is nurtured, particularly if their behavior appears sincere (Schoorman et al., 2007; Grandey et al., 2012).

On the other hand, previous research (Son et al., 2014; Qi et al., 2023) has illustrated Interactional Justice (IJ) moderates the association between leadership and several organizational outcomes such as employee engagement (Khan et al., 2018), as the quality of the social exchange relationship will affect the influence game of leadership and employees’ reciprocal behaviors (Cropanzano and Mitchell, 2005). Therefore, we propose the following hypothesis:

*H5b*: Interactional Justice (IJ) moderates the relationship between Authentic Leadership (AL) and Affective Commitment (AC).

**Figure 1:**
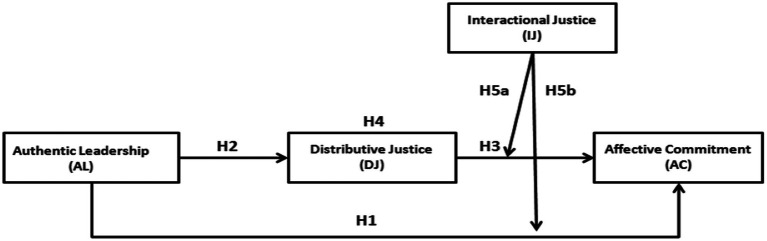
Research model.

A summary of each hypothesis and the relationships between the study variables is presented in Figure 1:

## 3 Materials and methodology

### 3.1 Participants and procedures

In order to achieve our objective, we conducted a research testing the proposed model’s hypotheses in workers from five retail companies in southern Spain. The study questionnaires clearly stated our research goals and protected participant anonymity. All who responded to the questionnaires explicitly agreed to participate. The entire study, involving human participants, followed the ethical guidelines of the Declaration of Helsinki and its revisions. No animals were involved in the study.

We distributed a total of 517 questionnaires, obtaining a response rate of 59.56%. Of the 305 completed questionnaires returned, three were excluded due to reasons like incomplete answers, choosing several options for a question or leaving some questions blank. Thus, the final study sample consisted of 302 workers.

About positions, they were from technical areas (55.3%), intermediate (21.85%), auxiliary (15.56%) and direction (7.28%) positions. The distribution by sex was balanced (56.62% females vs. 43.38% males) while the age ranges were broken down into 8.94% between 18 and 25; 25.82% between 26 and 35; 33.11% between 36 and 45; 24.50% between 46 to 55; and 7.62% over 56 years of age. Finally position tenure, in most cases, was less than 6 years (57.6%).

### 3.2 Questionnaire development and instruments

For the study, we designed a questionnaire which included the following tests:

For the measurement of Distributive and Interactional Justice, we used the corresponding dimensions of the Niehoff and Moorman Scale (Niehoff and Moorman, 1993) in its Spanish adaptation by Patlán Pérez et al. (2014). The Distributive Justice dimension consisted of five items related to workers’ perception of the results assigned to them in terms of remuneration or labor (e.g., “I believe that my job responsibilities are fair”). On the other hand, the Interactional Justice subtest included nine items and measured the degree to which employees perceive that they are treated equally, honestly and amicably (e.g., “When decisions are made about my work, my boss treats me with kindness and consideration”).

To measure the level of Affective Commitment the study resorted to the Affective Commitment Scale of Meyer and Allen (1997), adapted to Spanish by Arciniega and González (2006) which has 6 items that reflect the degree of identification and positive feelings of an individual concerning an organization (p.e “I feel like part of a family in this company”).

To measure Leadership, we used the Authentic Leadership Questionnaire ALQ (follower version) developed by Walumbwa et al. (2008) and adapted to Spanish sample by Moriano et al. (2011). This questionnaire included 16 items (e.g., “Making decisions based on values that matter”) in four dimensions: transparency in relationships (leader’s ability to be open and generate a climate of trust among followers); internalized moral perspective (which refers to a leader’s self-regulation based on personal beliefs and values); balanced information processing (leader’s ability to make decisions, objectively assess and evaluate available information); and leader self-awareness (leader’s knowledge of his or her strengths and weaknesses). In our study, to obtain an Authentic Leadership composite score, we adopted the methodology suggested by Luthans et al. (2008). Thus, first the values of the items that serve to evaluate each of the four dimensions were calculated to obtain a composite mean for each follower. In this sense, higher scores represent a higher perception of Authentic Leadership.

Responses in all instruments were collected through a five-point Likert-type scale where 1 represents “absolutely disagree” and 5 means “strongly agree,” with the exceptions of authentic leadership scale, where 1 means “never” and 5 signifies “always.”

Lastly, the questionnaire included additional sociodemographic questions (gender, age, job category and seniority).

### 3.3 Data analysis

To validate our working hypotheses, we conducted various statistical analyses on the collected dataset.

Firstly, we computed descriptive statistics, including measures of central tendency, dispersion, and skewness, utilizing the statistical software SPSS © v.25.

Subsequently, for the Partial Least Squares (PLS) analysis, we utilized the statistical package Smart PLS© v.4. The PLS-SEM analysis consists of two primary phases: the validation of a measurement model and the structural model.

Thus, to test the measurement model, in which the latent structure of the constructs and their respective indicators are analyzed, we verified the reliability of the constructs by calculating Cronbach’s Alpha coefficients and Composite Reliability (CR). Likewise, we evaluated convergent validity through factor loadings and Average Variance Extracted (AVE). Finally, we took as a reference the criterion established by Fornell and Larcker (1981) to test discriminant validity.

Finally, we validated the structural model by analyzing the structural relationships between the endogenous variables. This methodology allowed us to estimate the variance explained by the model, the magnitude of the effect as well as the statistical significance of the coefficients associated with the pathways that make up the model (Hwang et al., 2020). In addition, this technique allowed to overcome the non-normal distributions of the data and to incorporate explanatory and predictive perspectives in the estimation of the proposed model (Hair et al., 2019).

## 4 Results

### 4.1 Reliability and validity of the study model: preliminary analysis

With the data collected, a first analysis was to examine the reliability and validity of the measurement instruments used in our research.

To test convergent validity, we conducted a study of the structure and factor loadings of each item included in the questionnaires used in the study. After this initial analysis, we decided to eliminate two items from the Authentic Leadership dimension (AL 4 “My manager or supervisor tells me the truth even if it is hard”; AL 11 “My manager or supervisor analyses the relevant data before reaching a decision”) and one item from the Affective Commitment dimension (AC 6 “I would be very happy to spend the rest of my working life in this company”) because their factor loadings were lower than 0.6 (Chin, 1998), thus validating the internal consistency of the remaining 33 items with their respective constructs (see Table 1).

**Table 1:** Outler loadings, AVE, Cronbach’s alpha, and CR.

Latent variables	Indicator	Outer loading	AVE	Alfa Cronbach	CR
Authentic leadership	AL1	0.712	0.589	0.946	0.952
	AL2	0.818			
	AL3	0.765			
	AL5	0.743			
	AL6	0.740			
	AL7	0.623			
	AL8	0.803			
	AL9	0.742			
	AL10	0.716			
	AL12	0.849			
	AL13	0.843			
	AL14	0.704			
	AL15.	0.820			
	AL16	0.834			
Distributive justice	DJ1	0.698	0.560	0.804	0.864
	DJ2	0.756			
	DJ3	0.722			
	DJ4	0.842			
	DJ5	0.717			
Interactional justice	IJ1	0.847	0.737	0.955	0.962
	IJ2	0.815			
	IJ3	0.863			
	IJ4	0.906			
	IJ5	0.842			
	IJ6	0.835			
	IJ7	0.869			
	IJ8	0.880			
	IJ9	0.867			
Affective commitment	AC1	0.866	0.697	0.892	0.920
	AC2	0.827			
	AC3	0.854			
	AC4	0.764			
	AC5	0.860			

CR, composite reliability; AVE, average variance extracted. Source (s): Authors’ own work.

Next, we analyzed the Cronbach’s Alpha, Composite Reliability (CR), and Average Variance Extracted (AVE). In this case, we chose to include the Composite Reliability coefficient as it provides a less biased estimate of reliability than Cronbach’s Alpha (Gotz et al., 2010). As shown in Table 2, the Cronbach’s Alpha values and the Composite Reliability values of the constructs included in the study surpass 0.7 (Hair et al., 2021). Lastly, we also obtained an AVE value exceeding 0.5, confirming that the construct explains at least 50% of the variance (Cheung et al., 2023).

**Table 2:** Means, standard deviation and correlation.

			Fornell-Larcker			
	*M*	SD	AC	DJ	IJ	AL
Affective commitment (AC)	3.08	0.976	**0.835**			
Distributive justice (DJ)	3.09	0.889	0.553**	**0.749**		
Interactional justice (IJ)	3.37	0.981	0.586**	0.621**	**0.859**	
Authentic leadership (AL)	1.51	0.501	0.556**	0.555**	0.817**	**0.768**

Square root of AVE on diagonal; correlations between constructs are shown below the diagonal; *p < 0.05. **p < 0.01. Source (s): Author’ own work.

Having assessed convergent validity by the recommendations of Henseler et al. (2016), we proceeded to evaluate the discriminant validity of the model, verifying the criterion formulated by Fornell and Larcker (1981). Discriminant validity refers to the condition in which two or more distinct concepts are not correlated with each other. According to this criterion, the square root of the AVE for each construct should exceed the correlations between that construct and all other constructs in the model. As observed in Table 2, the elements on the main diagonal representing the square root of the AVE for each construct are greater than the correlations of the constructs as depicted in the row or column values. This demonstrates that the scales used in this study do not exhibit issues with discriminant validity.

Finally, we examined the potential risk of Common Method Bias (CMB) through Variance Inflation Factors (VIFs). As demonstrated in Table 3, the values obtained for the instruments in our study were below 5, confirming the proposed model did not have a collinearity problem.

**Table 3:** Full collinearity analysis.

	AC	DJ	IJ	AL
Affective commitment (AC)				
Distributive Justice (DJ)	1.738			
Interactional Justice (IJ)	3.825			
Authentic Leadership (AL)	3.193	1.000		

Source(s): Authors’ own work.

### 4.2 Structural model assessment

Once we had validated the instruments, we proceeded to evaluate our study model. For this purpose, we assessed the direct and indirect effects of each exogenous variable on the endogenous variable using a bootstrapping method with 5,000 resamples.

Regarding the direct effects, Table 4 shows that the variable Authentic Leadership (AL) presents a strong and statistically significant direct effect on Affective Commitment (AC) and Distributive Justice (DJ) which would support our Hypotheses 1 and 2.

**Table 4:** Structural model.

Relationship	β		BCI LL	BCI UL	*T* –value	*F* ^2^	Decision
*Direct effects*							
H1 AL- > AC	0.253**		0.088	0.419	4.738	0.035	Supported
H2 AL - > DJ	0.571**		0.488	0.646	13.894	0.475	Supported
H3 DJ- > AC	0.242**		0.124	0.355	4.056	0.064	Supported
*Mediating effect of distributive justice*							
H4 AL- > DJ- > AC	0.138**		0.069	0.212	3.767		Supported
*Moderating effect of interactive justice*							
H5a: IJ*DJ- > AC	–0.131**		−0.131	−0.227	1.085		Partial
H5b: IJ*AL- > AC	0.059		−0.055	0.166	2.737		Not supported

Al, Authentic leadership; DJ, distributive justice; IJ, interactional justice; AC, affective commitment; *p < 0.05. **p < 0.01; BCI UL, bias confidence interval upper level; BCI LL, bias confidence interval lower level. Source (s): Author’ own work.

Moreover, the variable Distributive Justice (DJ) had a statistically significant direct effect on Affective Commitment (AC) therefore we could support our Hypothesis 3.

Regarding the Hypothesis 4 in which we pointed out the mediating effect of Distributive Justice in these variables (Authentic Leadership and Affective Commitment), we could prove the data obtained.

Additionally, an adjustment of in the relationship between DJ and AC was also confirmed. Lastly, we could not demonstrate our H5b, as the connection is not substantially adjusted by IJ.

About the moderating effect from Interactional Justice (IJ), we obtained a negative and significant effect which would partially prove our Hypothesis 5a However, we could not obtain a statistically significant moderating effect between the variables Authentic Leadership (AL) and Affective Commitment (AC) therefore we could not support our Hypothesis 5b.

In Table 4 we also can observe the effect size of each variable concerning the hypotheses (*f*^2^), using the threshold proposed by Cohen (2013) in where values of 0.02, 0.12, and 0.35 indicate small, medium, and large effect sizes, respectively. The data obtained indicated that while both AL and DJ have a small effect on AC, AL does have a large effect on DJ.

**Figure 2:**
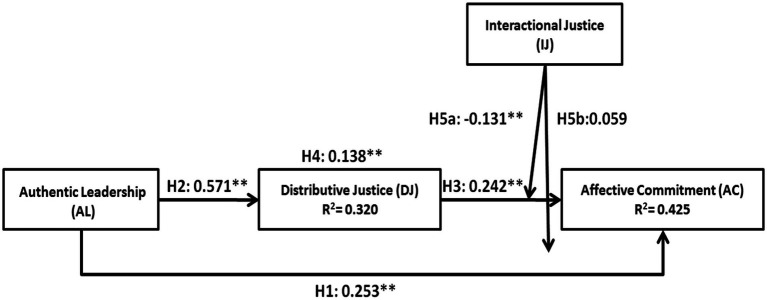
Results obtained in the study model. *p < 0.05. **p < 0.01. Source (s): Author’ own work.

A summary of the values obtained for the interaction of each variable, as well as the study hypotheses, can be observed in Figure 2.

This figure visualizes both the representation of the path coefficients between predictor and endogenous variables of the proposed model and their explanatory power (R^2^) for each of the relationships. Additionally, following the recommendations of Fornell and Larcker (1981), we estimated the Goodness-of-Fit (GoF) by calculating the square root of the product of the average *R*^2^ of the internal construct and the average AVE of the external construct. According to authors such as Wetzels et al. (2009), the adequacy of the structural model is sufficient when GoF > 0.36. Using this threshold as a reference and given that the estimated GoF for the structural model of the study is 0.4904, it demonstrates that the model fit is satisfactory.

## 5 Discussions and conclusion

### 5.1 Discussion of findings

Our study allowed us to have a more comprehensive view of the importance of the Authentic Leadership on the Affective Commitment and how this relationship in influenced by the perceived organizational justice.

The results of our study offer an evidence that leader serves as a tangible, rather than abstract incarnation of the image of an organization that is key to the link between employees and the organization. In this sense, the leader/follower relationship is crucial to grasp the influence of supervisors on employee organizational commitment (Wallace et al., 2013; Bohrt and Bretones, 2018).

In that sense, our study reveals the need for organizations to recruit and select leaders with authentic leadership competencies. Organizations must moreover focus on training and coaching to increase them as this can foster bolstering worker identification with an organization. In this sense, a low level of affective commitment can also serve as indicator of the necessity to consider replacing a supervisor.

Another variable that influences the development of affective commitment is the distributive justice. Our finding corroborates the results of previous research (Colquitt et al., 2013; Kassim and Ibrahim, 2016). This link can be attributed to the fact that distributive justice is associated with the concept of the psychological contract, whereby this dimension of justice strengthens the belief that the organization fulfills its part of the contract, thereby reinforcing the employee’s willingness to go beyond minimal job expectations and, consequently, their commitment to the organization. Another reason may be that in perceived fair environments, employees tend to develop a stronger sense of belonging and identification with their organization. Thus, this identification enhances affective commitment because employees not only value their relationship with the organization in contractual or economic terms but also as an integral part of their social and professional identity.

Furthermore, our study has been able to confirm the mediating role of distributive justice regarding the relationship between authentic leadership and affective commitment, as hypothesized from the results of previous studies. In this regard, we suggest that the perception of equal treatment in the distribution of resources acts as a moderator in the perception of integrity in leadership within the organization, thereby influencing the employee’s sense of belonging to their organization as a feeling of reciprocal value.

Regarding the effect of interactional justice, and contrary to the expected effect, it negatively moderated the relationship between distributive justice and affective commitment. One possible explanation is that employees may perceive interactional justice as a more immediate and tangible indicator of the organizational climate. Therefore, even if distributions were equitable, poor interpersonal behavior could be interpreted as a lack of consideration for their worth, leading to less emotional dedication. Another possible explanation is that while allocated resources may satisfy material wants, respectful interactions influence how valued and respected someone feels on a personal level. Thus, if fair treatment during interactions is seen as low, it could counteract the positive effects of equitable resource distributions, ultimately resulting in less affective commitment.

In contrast to numerous previous studies (Suifan, 2019; Thompson et al., 2021) that confirm the relationship between interactional justice and affective commitment, the second moderation proposed in the model between authentic leadership and affective commitment was not significant. This could be attributed that interactional justice and authentic leadership may influence affective commitment through different and parallel mechanisms. While authentic leadership enhances affective commitment through a shared vision and alignment of values, interactional justice may influence other aspects of employee well-being or satisfaction that do not necessarily modulate the impact of authentic leadership on commitment. In any case, these findings corroborate some of the results found in other studies, such as the one conducted by Jayus (2021).

### 5.2 Implications

This study makes several important implications for research and knowledge. First and foremost, this study reinforces the theory of authentic leadership by demonstrating its direct and significant impact on affective commitment, emphasizing the relevance of authentic characteristics in leaders for the development of organizational commitment. Furthermore, by highlighting the mediating role of distributive justice, it adds a new dimension to the theory of authentic leadership, suggesting that its effectiveness is not solely based on authenticity *per se* but also on how this authenticity translates into fair organizational practices. Lastly, the negative moderation of interactional justice underscores the complexity of interpersonal relationships in the realm of authentic leadership, suggesting that the effectiveness of an authentic leader may be compromised by potential deficiencies in everyday interpersonal interactions. These conclusions allow for an expanded understanding of the mechanisms through which authentic leadership influences, providing a more robust foundation for reference in future research and practices related to organizational leadership.

Furthermore, as evidenced by the results obtained in this study, the relationship between the supervisor and the employee plays a key role in how employees see distributive justice within their organization (Colquitt et al., 2013). Consistent with previous research (Kurian and Nafukho, 2022), authentic leadership has been shown to be effective in fostering the perception of equitable distribution among employees. This relationship may be attributed to its transparency and honesty, both of which can contribute to employees perceiving decision-making processes as fairer, subsequently influencing their perception of fairness in the distribution of rewards and workloads.

On the other hand, the negative moderation of interactional justice suggests that even in the presence of a fair distribution of resources and authentic leadership, poor interpersonal treatment can diminish affective commitment. This implies that organizations must pay attention not only to distribution policies and practices but also to the quality and nature of daily interactions between supervisors and employees.

Finally, given that interactional justice did not significantly moderate the relationship between authentic leadership and affective commitment, organizations should consider these two elements as independent yet complementary avenues for enhancing employee commitment.

### 5.3 Limitations and future research directions

While the findings of our study are highly valuable and promising, we acknowledge certain limitations that should be considered in future research.

One such limitation is that although the number of participating workers in the study was adequate, we believe that future research should seek to apply our findings to other national samples and in different economic sectors, to contrast the data obtained in our study.

Moreover, it would be advisable in future research to carry out longitudinal studies that more precisely explore leadership as a social process.

Furthermore, future research should continue to examine the impact of diverse leadership approaches, such as transformational leadership or inclusive leadership, to compare their influences on employee commitment, following previous studies such as those developed by Abasilim et al. (2019), who analyzed the relationship among different leadership styles (transformational, transactional and *laissez-faire* leadership) and employee engagement, or Jiatong et al. (2022), who showed that transformational leadership had a positive impact on affective commitment to the organization and job performance of employees in the hotel industry.

Lastly, the findings of this research underscore that leader behavior has a significant effect on both employee commitment and perceptions of justice. However, we suggest that the future research include the impact of additional variables and their potential mediating role in the relationship between these two variables.

## Data availability statement

The raw data supporting the conclusions of this article will be made available by the authors, without undue reservation.

## Ethics statement

The study questionnaires clearly stated our research goals and protected participant anonymity. All who responded to the questionnaires explicitly agreed to participate. The entire study, involving human participants, followed the ethical guidelines of the Declaration of Helsinki and its revisions.

## Author contributions

AG-C: Conceptualization, Resources, Software, Writing – original draft, Writing – review & editing. AT: Conceptualization, Formal analysis, Methodology, Writing – original draft, Writing – review & editing. FB: Data curation, Project administration, Supervision, Validation, Writing – original draft, Writing – review & editing. JF-M: Formal analysis, Investigation, Methodology, Visualization, Writing – original draft, Writing – review & editing.
